# DeepReg: a deep learning hybrid model for predicting transcription factors in eukaryotic and prokaryotic genomes

**DOI:** 10.1038/s41598-024-59487-5

**Published:** 2024-04-21

**Authors:** Leonardo Ledesma-Dominguez, Erik Carbajal-Degante, Gabriel Moreno-Hagelsieb, Ernesto Pérez-Rueda

**Affiliations:** 1 Posgrado en Ciencia en Ingeniería de la Computación, Universidad Nacional Autónoma de México, 04510 Mexico City, Mexico; 2 Instituto de Investigaciones en Matemáticas Aplicadas y en Sistemas, UNAM, 04510 Mexico City, México; 3 Coordinación de Universidad Abierta, Innovación Educativa y Educación a Distancia (CUAIEED), Universidad Nacional Autónoma de México, 04510 Mexico City, México; 4 Department of Biology, Wilfrid Laurier University, Waterloo, ON Canada; 5 Instituto de Investigaciones en Matemáticas Aplicadas y en Sistemas, Unidad Académica del Estado de Yucatán, Universidad Nacional Autónoma de México, Mérida, Yucatán México

**Keywords:** Computational biology and bioinformatics, Protein analysis, Protein function predictions

## Abstract

Deep learning models (DLMs) have gained importance in predicting, detecting, translating, and classifying a diversity of inputs. In bioinformatics, DLMs have been used to predict protein structures, transcription factor-binding sites, and promoters. In this work, we propose a hybrid model to identify transcription factors (TFs) among prokaryotic and eukaryotic protein sequences, named Deep Regulation (DeepReg) model. Two architectures were used in the DL model: a convolutional neural network (CNN), and a bidirectional long-short-term memory (BiLSTM). DeepReg reached a precision of 0.99, a recall of 0.97, and an F1-score of 0.98. The quality of our predictions, the bias-variance trade-off approach, and the characterization of new TF predictions were evaluated and compared against those produced by DeepTFactor, as well as against experimental data from three model organisms. Predictions based on our DLM tended to exhibit less variance and bias than those from DeepTFactor, thus increasing reliability and decreasing overfitting.

## Introduction

DNA-binding transcription factors (TFs) play a fundamental role in modulating the expression of specific genes, depending on metabolic requirements. Control of gene expression in all organisms occurs by the binding of these proteins to specific sites upstream, downstream, or overlapping the promoter^1^. For instance, in bacteria, most repressors block the promoter, making it inaccessible to RNA polymerase, whereas activators enhance RNA polymerase binding^2^. In eukaryotic organisms, similar mechanisms have been described, with the most substantial difference being the formation of TF multimeric complexes^3^. TFs link signal flow and gene expression. However, their functionality depends on many environmental conditions, and their involvement in a particular signaling pathway is sometimes difficult to predict. Therefore, the presence of specific TF types can provide information about the possible existence of signaling pathways, or, in the opposite case, the absence of certain TF types could indicate the absence of the corresponding pathway^4^.

With the advent of the genomic era, many organisms have been sequenced, and their protein repertoire has been elucidated, making it possible to explore proteins with specific functions, such as enzymes, transporters, and TFs. In contrast, experimental evidence is limited compared to the number of gene and protein sequences available. For instance, less than 1000 bacterial TFs with direct experimental evidence are available thus far, whereas the function of more than 50,000 proteins has been inferred by sequence comparisons^5–7^, implying a bias against the discovery of TFs that belong to families that have not previously been experimentally explored. Relatively few works address the prediction of TFs using deep learning (DL) algorithms, such as DeepTFactor^8^, and TFpredict^9^. The use of DL models for the prediction and classification of genomic data, such as gene expression data, as well as the analysis of large amounts of sequence information, is increasing in the last years. In this regard, DL algorithms have shown great performance in the identification of proteins with diverse functions, such as transport^10^, enzymes^11^, and regulatory roles^8^. DeepTP identifies putative transporter proteins by considering a convolutional neural network model that uses parallel subnetworks to extract features from protein sequences, adding fully connected layers for TP classification. DeepTP achieved better performance than other commonly used methods and made predictions by recognizing the functional domains of TPs^11^. Alternatively, DeepEC uses 3 convolutional neural networks (CNNs) for the prediction of EC numbers, combined with homology analysis inferring EC numbers that cannot be classified by the CNNs^11^. Finally, a DL model based on self-attention and a multiple-channel feature fusion, called DeepTP, has been recently proposed to predict thermophilic proteins. To do this, a CNN bidirectional long short-term memory network was used to extract hidden features in protein sequences. Different weights were then assigned to features through self-attention, and, finally, biological features were integrated to build a prediction model. DeepTP had better performance and scalability in an independent balanced test set and validation set^12^.

In addition, DL has been used to analyze the characteristics of eukaryotic promoter sequences for the accurate recognition of human and mouse promoter sequences^13,14^, for de novo prediction of noncoding functions^15,16^, for protein classification^17–19^ and, more recently, for the prediction of protein structures with atomic accuracy, even in cases in which no similar structure is known^20^.

Transcription factors (TFs) represent an excellent group of proteins to be analyzed and identified by DL, where DL might overcome limitations of sequence comparisons. To address this challenge, we constructed a DL architecture to improve the identification of TFs in prokaryotic and eukaryotic organisms, based on a training set of TFs, extracted from Swissprot^21^. The best model was selected by Regularization, Early Stopping, and Learning Rate Schedule to avoid overfitting without compromising performance. Additionally, better performance of DL models against machine learning approaches using support vector machines, random forest, logistic regression, Gaussian NB, and another DL model was evidenced. Later, a comparative analysis of TFs in three organisms (*Saccharomyces cerevisiae, Aspergillus nidulans,* and *Neurospora crassa*) was assessed for their effectiveness in the identification of experimentally characterized TFs. Finally, a metric approach to measure the quality of predictions by bias-variance trade-off is proposed.

## Methods

### Database construction with information on TFs

**Figure 1:**
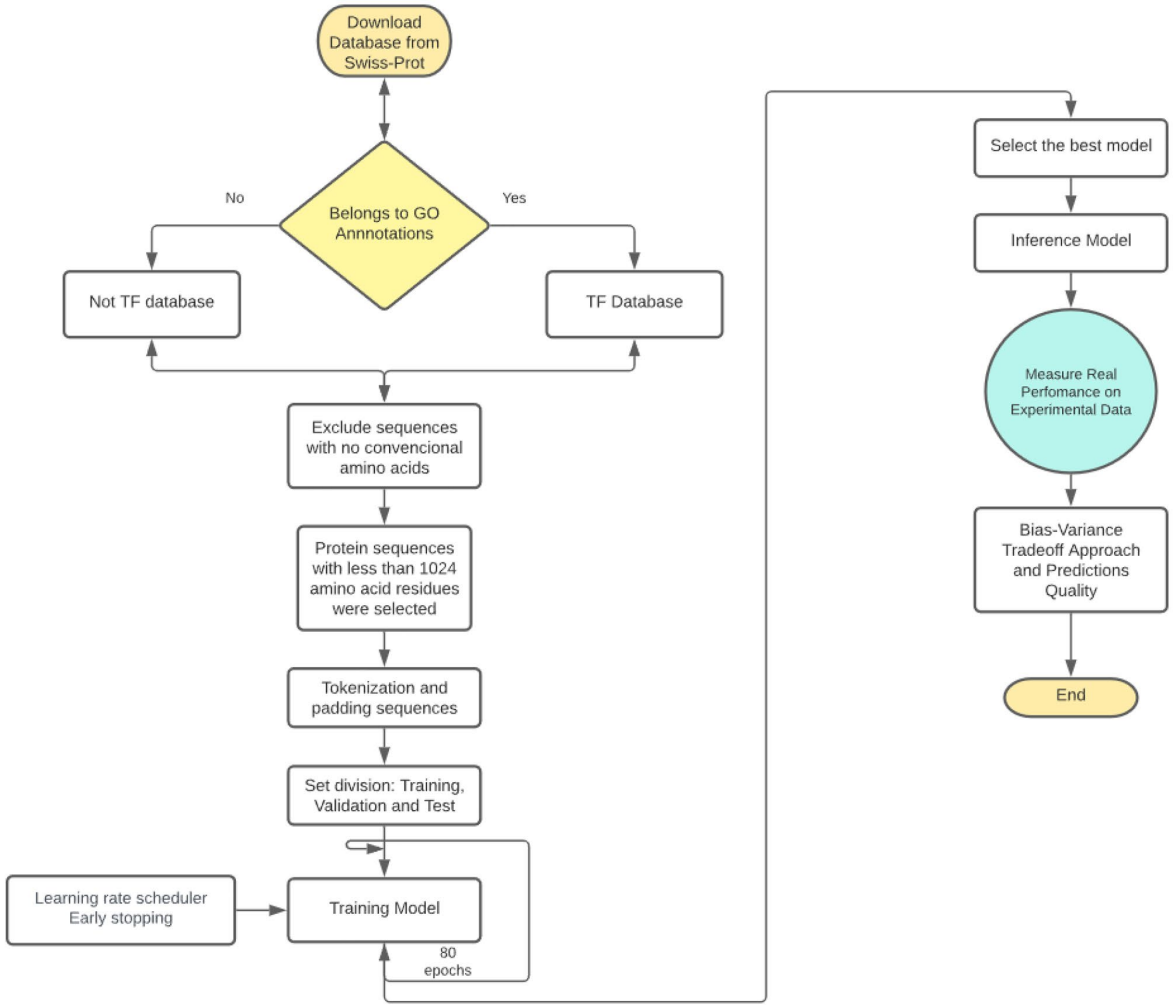
Flow diagram of the DL used to predict transcription factors. A total of 22,100 TFs and 527,146 non-TFs were retrieved from SwissProt. These sequences were cleaned to be tokenized and padded. In a posterior step, the training, validation, and test datasets were built at a ratio of 90:9:1, in that order. The model was trained for a maximum of 80 epochs using a learning rate scheduler and early stopping to avoid overfitting. After many trial and error rounds; changing hyperparameters, such as batch size, dropout rates, and initial learning rates. Finally, an inference model was produced to evaluate performance and quality against experimental data using a bias-variance tradeoff.

To construct a dataset of transcription factors (TFs), we retrieved UniProtKB (Reviewed SwissProt) (March 2022)^21^. We identified 36 Gene Ontology (GO) terms associated with TFs, grouped in the following categories: “transcription regulatory region sequence-specific DNA binding”, “positive regulation of DNA-binding, initiation”, “negative regulation of DNA-binding, initiation”, and “DNA-binding transcription factor activity” (Table S2). In addition, only protein sequences from the reviewed SwissProt database with less than 1024 amino acid residues were selected, while sequences with unconventional amino acids were removed. In total, 22,100 protein sequences identified as TFs, and 527,146 non-TF sequences were considered for the analysis. Finally, to consider a ratio of 5:1 between negative and positive samples, 18,415 TF and 92,085 non-TF sequences were randomly selected (Fig. 1).

### Deep learning techniques and architectures

Two specific architectures were used in the DL model: a convolutional neural network (CNN), which works as a feature extractor, with four parallelized layers and different filter sizes, as previously reported^8^. Those filter sizes were used for identifying patterns with different receptive fields, working as variable windows to scan amino acid sequences. The second architecture considers a bidirectional long-short-term memory (BiLSTM) designed to predict a sequence at time instance t + 1 based on a sequence at time instance t. The LSTM architecture constructs a contextual grammar by processing tokenized sequences as input.

For instance, given the input sequence* I* of amino acids of length *n*, let the residue be *X* at position *k* from *I*, where *k* is a position, such that *k* ≤ *n*, the LSTM process is defined as:

$${\varvec{X}},\boldsymbol{ }{{\varvec{X}}}^{\boldsymbol{^{\prime}}}{\varvec{\epsilon}}\boldsymbol{ }{\varvec{I}},$$

$${BiLSTM(X)}_{k}={ X{\prime}}_{k+1},$$

where X’ is the predicted residue at time k + 1.

In addition, we used the traditional loss function for a binary cross-entropy classification that quantifies the closeness of the predictions of a newly trained model against the input data labels of those predictions; thus, the model is robust if the loss function tends to zero.

To exclude overfitting, the Early Stopping and Learning Rate Scheduler techniques were used. Both techniques used a hyperparameter called “patience”, which checks, within several epochs, if there is a continuous improvement in the loss function; otherwise, early stopping stops, and the learning rate decreases, fitting the weights of the model. In addition, we used a hybrid regularization technique between L1 (Lasso) and L2 (Ridge) called ElasticNet and the dropout technique.

Therefore, taking the amino acid sequences as input, ElasticNet regularized the model considering the hypothesis that there is no correlation in the amino acid sequence (L1 Regularization) *versus* the opposite case, i.e. considering that there is a correlation (L2 Regularization). ElasticNet allows us to generalize the model and avoid overfitting. Finally, the dropout technique was used as regularization and as an approximation to the Bayesian uncertainty model^22^, which allows for increased reliability.

### DeepReg model

The model proposed in this work, which we called Deep Regulation (DeepReg), considers, as the first instance, the original model described in DeepTFactor^8^. However, an additional CNN and an LSTM net at the end of the connected CNNs were included to improve performance. The CNN networks obtain the descriptive features of the input data and convert them into time series to be posteriorly used by the LSTM. Therefore, three datasets with a proportion of 90:9:1 for training, validation, and testing were selected. It is worth mentioning that, unlike previous works, a test process was carried out to measure the performance at the end of the model, as well as to calculate the confusion matrix to quantify the behavior of the model against experimental data.

### Tokenization, padding, and one-hot encoding

In the first step, all protein sequences were tokenized, where each amino acid took only one number value. This tokenization is important to make the data more interpretable by any DL model. For instance, the following 473 long sequence of amino acid residues:

“MGRKKIQITRIMDERNRQVTFTKRKFGLMKKAYELSVLCDCEIALIIFNSTNKLFQYASTDMDKVLLKYTEYNEPHESRTNSDIVETLRKKGLNGCDSPDPDADDSVGHSPESEDKYRKINEDIDLMISRQRLCAVPPPNFEMPVTIPVSSHNSLVYSNPVSSLGNPNLLPLAHPSLQRNSMSPGVTHRPPSAGNTGGLMGGDLTSGAGTSAGNGYGNPRNSPGLLVSPGNLNKNIQAKSPPPMNLGMNNRKPDLRVLIPPGSKNTMPSVSEDVDLLLNQRINNSQSAQSLATPVVSVATPTLPGQGMGGYPSAISTTYGTEYSLSSADLSSLSGFNTASALHLGSVTGWQQQHLHNMPPSALSQLGACTSTHLSQSSNLSLPSTQSLNIKSEPVSPPRDRTTTPSRYPQHTRHEAGRSPVDSLSSCSSSYDGSDREDHRNEFHSPIGLTRPSPDERESPSVKRMRLSEGWAT”.

was tokenized into 473 numerical values:

[11, 6, 15, 9, 9, 8, 14, 8, 17, 15, 8, 11, 3, 4, 15, 12, 15, 14, 18, 17, 5, 17, 9, 15, 9, 5, 6, 10, 11, 9, 9, 1, 4, 10, 16, 18, 10, 2, 3, 2, 4, 8, 1, 10, 8, 8, 5, 12, 16, 17, 12, 9, 10, 5, 14, 1, 16, 17, 3, 11, 3, 9, 18, 10, 10, 9, 17, 4, 12, 4, 13, 7, 4, 16, 15, 17, 12, 16, 3, 8, 18, 4, 17, 10, 15, 9, 9, 6, 10, 12, 6, 2, 3, 16, 13, 3, 13, 3, 1, 3, 3, 16, 18, 6, 7, 16, 13, 4, 16, 4, 3, 9, 15, 9, 8, 12, 4, 3, 8, 3, 10, 11, 8, 16, 15, 14, 15, 10, 2, 1, 18, 13, 13, 13, 12, 5, 4, 11, 13, 18, 17, 8, 13, 18, 16, 16, 7, 12, 16, 10, 18, 16, 12, 13, 18, 16, 16, 10, 6, 12, 13, 12, 10, 10, 13, 10, 1, 7, 13, 16, 10, 14, 15, 12, 16, 11, 16, 13, 6, 18, 17, 7, 15, 13, 13, 16, 1, 6, 12, 17, 6, 6, 10, 11, 6, 6, 3, 10, 17, 16, 6, 1, 6, 17, 16, 1, 6, 12, 6, 6, 12, 13, 15, 12, 16, 13, 6, 10, 10, 18, 16, 13, 6, 12, 10, 12, 9, 12, 8, 14, 1, 9, 16, 13, 13, 13, 11, 12, 10, 6, 11, 12, 12, 15, 9, 13, 3, 10, 15, 18, 10, 8, 13, 13, 6, 16, 9, 12, 17, 11, 13, 16, 18, 16, 4, 3, 18, 3, 10, 10, 10, 12, 14, 15, 8, 12, 12, 16, 14, 16, 1, 14, 16, 10, 1, 17, 13, 18, 18, 16, 18, 1, 17, 13, 17, 10, 13, 6, 14, 6, 11, 6, 6, 13, 16, 1, 8, 16, 17, 17, 6, 17, 4, 16, 10, 16, 16, 1, 3, 10, 16, 16, 10, 16, 6, 5, 12, 17, 1, 16, 1, 10, 7, 10, 6, 16, 18, 17, 6, 19, 14, 14, 14, 7, 10, 7, 12, 11, 13, 13, 16, 1, 10, 16, 14, 10, 6, 1, 2, 17, 16, 17, 7, 10, 16, 14, 16, 16, 12, 10, 16, 10, 13, 16, 17, 14, 16, 10, 12, 8, 9, 16, 4, 13, 18, 16, 13, 13, 15, 3, 15, 17, 17, 17, 13, 16, 15, 13, 14, 7, 17, 15, 7, 4, 1, 6, 15, 16, 13, 18, 3, 16, 10, 16, 16, 2, 16, 16, 16, 3, 6, 16, 3, 15, 4, 3, 7, 15, 12, 4, 5, 7, 16, 13, 8, 6, 10, 17, 15, 13, 16, 13, 3, 4, 15, 4, 16, 13, 16, 18, 9, 15, 11, 15, 10, 16, 4, 6, 19, 1, 17].

The tokenized sequences were padded to the same length, i.e. we used a padding size of 1000 elements and applied one-hot encoding. As a result, a tensor *n* × *1000* × *20,* where *n* is the number of sequences, was considered.

## Results

### DeepReg architecture

**Figure 2:**
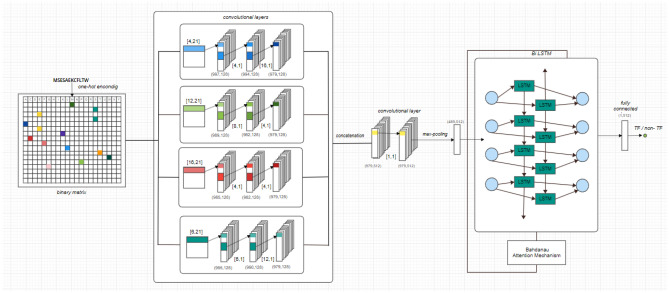
The network architecture of DeepReg. Three modules were considered: (1) 4 CNN layers as the feature extractor, (2) LSTM, and (3) the attention mechanism module. Each sequence is tokenized and passes through a one-hot encoding process. See the text for details.

To identify transcription factors (TFs) in prokaryotic and eukaryotic genomes, we propose a hybrid model called Deep Regulation (DeepReg) that involves a quadruple convolutional neural network connected to a bidirectional long short-term memory network organized into three modules (Fig. 2):

$$ \begin{gathered} O_{{CNN_{n} }} = Conv2D\left( {Conv2D\left( {Conv2D\left( I \right)} \right)} \right) \hfill \\ O_{4CNN} = MaxPool\left( {Conv1D\left( {Concat\left( {O_{{CNN_{1} }} ,\,O_{{CNN_{2} }} ,O_{{CNN_{3} }} ,O_{{CNN_{4} }} } \right)} \right)} \right). \hfill \\ \end{gathered} $$

- A module with four convolutional neural networks (CNNs) with four different filter sizes: [4, 4, 16] and [6, 6, 12] as upsampling operations; and [4, 8, 12] and [4, 4, 16] as downsampling operations. Upsampling and downsampling operations provide different features in different receptive fields of sequences. The output of the 4 CNN modules was calculated as follows:
  where Conv2D and Conv1D are convolution processes in 2D and 1D, respectively; Concat is a concatenation, and MaxPool is max pooling.
- BiLSTM module. To apply max pooling to the 4 CNN module outputs, we used BiLSTM with 128 units connected with an attention mechanism to select the best features from the TF and non-TF datasets. The LSTM network predicts a future subsequence from an input subsequence, determining a series of relevant features.
- Attention Mechanism (AM): DeepReg used the Bahdanau attention mechanism^23^ to improve the model focus on relevant parts of the input sequence after new predictions. In brief, AM works by assigning a weight to each input element based on its relevance to the current prediction. Subsequently, those weights are used to compute a weighted sum of the input elements, which is sent to the BiLSTM. This procedure allows selection of the most relevant parts of the input sequence, improving the model’s ability to handle long sequences and capture complex patterns.

Finally, BiLSTM with the AM module output is calculated by:

$$ O_{BiLSTM} = AM\left( {LSTM_{forward} \left( {O_{4CNN} } \right),LSTM_{backward} \left( {O_{4CNN} } \right)} \right). $$

The final output follows the equation:

$$ O_{{Deep{\text{Re}} g}} = LR\left( {R_{ElasticNet} \left( {FC\left( {O_{BiLSTM} } \right)} \right)} \right), $$

where *LR* represents the learning rate, *R,* ElasticNet regularization; and *FC* is fully connected.

### Training and performance model

To avoid overfitting and obtain the best possible model, we selected the model with the lowest value of the loss function and a learning rate after 10 out of 80 epochs with no decrease in performance. Additionally, 5 epochs were assessed to evaluate the loss function behavior and oscillations. Therefore, the best model was found at 30 epochs, prompting the training process to conclude at 35 epochs due to the absence of substantial improvement.

The algorithm was optimized with adaptive moment estimation (ADAM), which is the common optimizer for classification tasks, considering a binary cross-entropy as the loss function. Each convolutional layer incorporated 128 filters, with rectified linear unit (ReLu) function activation. Therefore, the following hyperparameters were considered: a learning rate scheduler with patience equal to 10, a minimum learning rate of 0.00001 with a factor of 0.1, and a learning rate start of 0.001. For early stopping, a value of 15 for the monitor on loss validation and patience was considered. Finally, a batch size of 128, and the dropout values of 0.3, 0.5, and 0.7 for each CNN layer were used.

**Figure 3:**
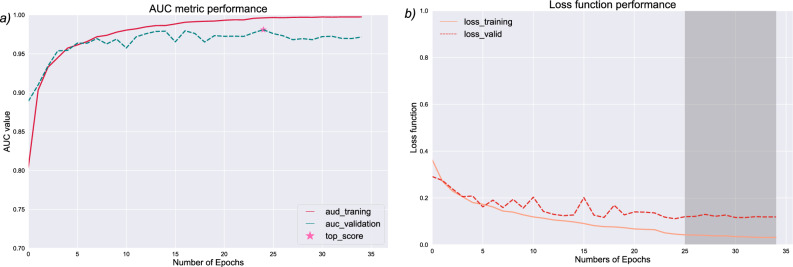
(**a**) AUC metric and (**b**) loss function of architecture performance. In (**a**), the X-axis shows the number of epochs against the value of the AUC and loss functions, and the top score value is represented with a star. In (**b**), the X-axis shows the number of epochs against the loss function, and the shaded area shows the stability of the stall function, avoiding abrupt jumps.

The loss function exhibited a continuous decrease until it reached a minimum according to the plateau criterion (Fig. 3). This behavior indicated a lack of abrupt fluctuations in the loss function throughout the training process. It is crucial to consider these observations when assessing the ability of the model to generalize to new data. Although a model may achieve satisfactory performance, if the loss function fluctuates with both downward and upward movements, it would suggest inherent overfitting, such as with DeepTFactor^8^.

Therefore, we selected the best model for comparison against DeepTFactor^8^. DeepReg reached a precision of 0.99, against 0.96 for DeepTFactor, the recall of DeepReg was 0.97 *versus* 0.94 for DeepTFactor, and the F1-score for DeepReg was 0.98 against 0.95 for DeepTFactor (Table 1). Accuracy is not a relevant measure for unbalanced classes because it does not reflect the real performance of the model. Sensitivity (or recall) (DeepReg: 0.9770, DeepTFactor: 9428) and specificity (DeepReg: 0.9591, DeepTFactor: 0.9888) were used to assess the capacity of the model to distinguish true TFs from non-TFs. Sensitivity focuses on accurately identifying positive cases, while specificity concentrates on correctly identifying negative cases. Similar results were found when, the datasets were split in an 8:1:1 ratio (training, validation, and test datasets), as was originally described for DeepTFactor; i.e. better values in three out five metrics, Precision, recall and F1-Score. See Table 1.

**Table 1:** Performance metrics from each model.

Metrics	DeepReg (8:1:1)	DeepReg (9:0.9:0.1)	DeepTFactor (8:1:1)
Accuracy	0.9557	0.9742	0.9773
Precision	**0.9819**	**0.9923**	0.9656
Recall	**0.9590**	**0.9770**	0.9428
Specificity	0.9454	0.9591	0.9888
F1-score	**0.9703**	**0.9846**	0.9541
MCC	0.8838	0.9066	0.9392

Significant values are in bold.

In addition, we evaluated the performance of the model by splitting the training dataset into eukaryotic and prokaryotic sequences. To this end, 10,893 TF and 171,310 non-TF sequences were considered for eukaryotic sequences, and 10,038 TF and 321,743 non-TF sequences were considered for prokaryotic sequences. From this analysis, the accuracy was found to be better for prokaryotes than eukaryotes (0.98 versus 0.96), whereas the F1 score was better for prokaryotes than eukaryotes (0.9485 versus 0.8855) Table S3.

In summary, the DeepReg model showed better performance in predicting TFs than DeepTFactor. In addition, it showed better performance in the identification of prokaryotic than eukaryotic TFs. The identification of TFs among prokaryotic and eukaryotic proteins could be associated with the diversity of DNA-binding domains in bacteria and archaea, where the helix-turn-helix (HTH) is the predominant structure, versus eukaryotes, where a plethora of DNA-binding domains have been identified, such as the HTH, the basic leucine‐zipper (bZIP) and the basic helix‐loop‐helix (bHLH)^24^, or the homeodomain or homeobox (HD/Hox), among others^25^.

### DL architecture against the ML approach

To compare the DeepReg model against other machine learning (ML) techniques, a binary classification problem was designed. An initial preprocessing stage was implemented to transform the original sequence data into a format compatible with ML algorithms. The key element of this data representation is the utilization of the bigram methodology, which embodies a probabilistic model enabling the estimation of two successive elements within a data sequence. Moreover, a set of methodologies, including Gaussian naïve Bayes, logistic regression, support vector machines (SVMs), random forest, and decision trees, were used. Drawing from a meticulously balanced dataset comprising 40,000 sequences, the data were randomly divided into 80% for training and 20% for validation purposes.

The cumulative findings reveal the efficacy of each methodology when applied to both complete sequences and instances where principal component analysis (PCA) reduction is used. Notably, SVM shows the best performance, achieving a maximum F1-score of 88.8% in instances where PCA is omitted. This was followed closely by logistic regression, which attained an F1-score of 80.5% Table 2. Similar trends were found when evaluating the AUC values for each classification experiment, where SVM outperforms its counterparts in scenarios excluding PCA Table 3. Indeed, PCA assumes a pivotal role in diminishing the dimensionality of data representation, facilitating expeditious analysis without significantly compromising classification accuracy.

**Table 2:** Performance of traditional ML techniques in terms of F1-score for TF/not TF classification.

F1-score	No PCA	PCA 2-elements	PCA 3-elements	PCA 4-elements
Gaussian NB	0.5656	0.5948	0.5281	0.5178
Logistic regression	0.8054	0.6096	0.6313	0.6179
SVM	0.8879	0.6611	0.6509	0.6688
Random forest	0.7534	0.6585	0.6732	0.6812
Decision tree	0.7994	0.6934	0.7298	0.7621

**Table 3:** Performance of traditional ML techniques in terms of AUC metric for TF/not TF classification.

AUC	No PCA	PCA 2-elements	PCA 3-elements	PCA 4-elements
Gaussian NB	0.6594	0.5641	0.5978	0.6146
Logistic regression	0.8016	0.6136	0.5477	0.6392
SVM	0.8862	0.6419	0.6797	0.6956
Random forest	0.7677	0.6563	0.6856	0.6992
Decision tree	0.7904	0.6848	0.7241	0.7534

Finally, to compare our method against ML techniques, we used the same dataset of 40,000 sequences with a ratio of 8:1:1 for training, validation, and test). From this comparison, we found that DeepReg works better than ML algorithms evaluated in all metrics. Indeed, all metrics considered show a better performance than the best ML model, Support Vector Machine (SVM), such as Accuracy and F1 Supplementary Table S4. The better performance obtained by DeepReg could be associated with the quantity of data to train the models. ML algorithms pursue the objective of solving a task based on less data than algorithms based on Deep Learning. Precisely because the ML objective is to find the explainability of the nature and distribution of the data. In contrast, the solution found in DL is more efficient if more data is available.

In summary, DeepReg not only improves metrics over traditional ML techniques but also shows remarkable scalability for the efficient handling of large data sequences.

### Ablation study

To determine the contribution of each module in DeepReg, an ablation study was performed, where we considered: (1) the original CNN proposed by DeepTFactor, here called baseline, (2) an extra convolutional layer to the baseline, which would be the fourth in our model, (3) the BiLSTM network, (4) and finally we measured the performance adding the attention mechanism.

The addition of a new CNN network to the baseline improves the performance of the model (Table 4); however, when we tried to connect other one CNN network, it did not improve it significantly, therefore we only considered 4 CNNs. In general, the metrics improve by using BiLSTM network by 0.4 in AUC metric and 0.5 in F1, whereas, adding Bahdanau attention mechanism, there is an increasing of 0.1 in AUC metric and 0.1 in F1 (3) evaluation.

**Table 4:** Ablation analysis of DeepReg.

Method	Accuracy	AUC	F1-score	Recall	Precision	MCC
(1) Baseline	0.895	0.859	0.932	0.900	0.968	0.704
(2) Baseline + 1 CNN	0.919	0.905	0.920	0.9258	0.9144	0.8384
(3) DeepReg – ATT	0.963	0.946	0.976	0.968	0.984	0.902
(4) DeepReg	**0.974**	**0.956**	**0.985**	**0.977**	**0.992**	**0.906**

(1) Baseline, (2) Addition of one more CNN network proposed in this work, (3) BiLSTM addition (2) DeepReg without attention mechanism, (4) all modules of DeepReg.

Significant values are in bold.

Additional techniques were explored to improve network performance without falling into overfitting, such as Data augmentation, with no improvement by using artificial sequences, and training using contact surfaces of TFs. In the last case, there was not much improvement since the contact surfaces are too small to be generalized around large sequences.

In summary, ablation analysis suggests high predictive quality derived from the innovations applied to the predictive inputs. As shown in Table 4, compared with the model of removing the attention mechanism, and when we consider one-hot encoding, the accuracy, F1-score, AUC and MCC were improved by 1.0%.

## Discussion

### Analysis of TFs with experimental evidence

**Figure 4:**
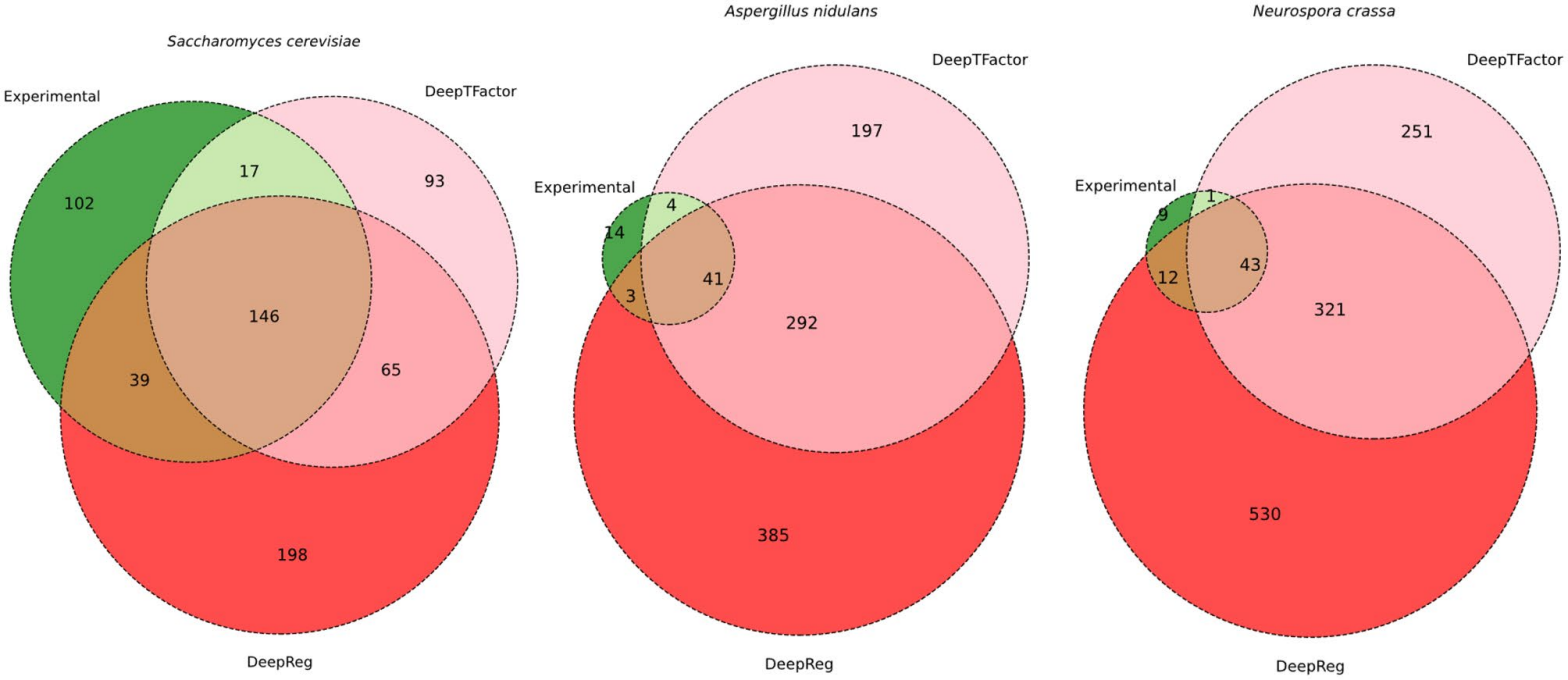
Efficiency of predictions associated with DeepReg and DeepTFactor. The dataset of TFs was predicted in three fungal models: *S. cerevisiae, N. crassa*, and *A. nidulans,* and compared against their repertoire of experimentally characterized TFs.

To determine the efficiency of predictions associated with DeepReg, the dataset of TFs was predicted in three fungal models: *S. cerevisiae, N. crassa*, and *A. nidulans,* and compared against their repertoire of experimentally characterized TFs, and those predictions made with DeepTFactor. In brief, 304 TFs experimentally characterized of *S. cerevisiae* were obtained from the YEASTRACT + database^26^, whereas the information for *A. nidulans* with 62 TFs and *N. crassa* with 75 TFs was collected from Hu et al.^27^. In Fig. 4, we show the predictions obtained with the two methods and their intersections concerning experimental information and those TFs predicted with DeepTFactor. We found that 163 and 185 out of 304 experimentally characterized TFs in *S. cerevisiae* were identified by DeepTFactor and DeepReg, respectively. In contrast, 102 TFs were not identified by either method, suggesting that 33.5% of the experimentally validated TFs were missed by both models. When we evaluated the prediction efficiency in *N. crassa,* we found that 46 and 44 out of 62 TFs were identified by DeepTFactor and DeepReg, respectively, whereas 14 out of 62 (22.5%) TFs were not identified by either method. Finally, in *A. nidulans*, 56 and 44 of 55 TFs were identified by DeepTFactor and DeepReg, respectively, and 9 (13.8%) were not found by either method. In addition, 356 new TFs were predicted in *S. cerevisiae*, and among these, 65 were predicted by both methods; 874 TFs in *N. crassa* were identified, with 292 identified by both methods, and 1102 TFs were predicted in *A. nidulans* (321 common to DeepTFactor and DeepReg).

In summary, DeepTFactor and DeepReg identified 53% and 60% of the experimental TFs in *S. cerevisiae*, 72.5% and 70.9% in *N. crassa*, and 67.6 and 84.6% in *A. nidulans*. In contrast, an average of 23.3% of the experimentally described TFs in the three models were not identified by either of the two methods. Therefore, based on the two approaches to identifying TFs, we missed (on average) 22% of potential TFs in all the fungal genomes*,* probably because some of these TFs are species-specific or they have noncanonical DNA-binding structures, such as the bromodomains associated with histone acetyltransferase and chromatin remodeling^28,29^ identified in six TFs (SWI/SNF or NP_014933.3, SAGA or NP_009637.1, RSC or NP_011570.1, NP_011570.1, Rsc2p or NP_013461.1, and NP_013461.1) or the DNA-binding domain of the Mlu1-box binding protein MBP1 of *A. nidulans* (rgdA or XP_660758.1 and XP_664319.1). Finally, the identification of new DNA-binding structures could improve TF predictions in these organisms.

In addition, to evaluate the quality of the predictions, i.e. the reliability and certainty of a model, a bias-variance tradeoff approach was proposed. This approach shows a tradeoff between bias and variance^30^, and it is used to detect overfitting and underfitting models^31^ to obtain a desirable model, minimizing bias and variance from its predictions. The approach is presented using a bull’s-eye diagram, where, if predictions are concentrated close to the center, the model is optimal, i.e. it has low variance and low bias. In contrast, if the predictions are scattered and fall far from the center, the model is overfitting, i.e. it has a high variance but low bias. Sometimes, predictions fall isolated and concentrated from the center, and the model has low variance but high bias, suggesting underfitting. Otherwise, if the predictions fall scattered and far from the center, the model is useless for solving the task. This means that the model has high bias and variance. In this regard, the range of true predictions goes from 0.50 to 1.0, which in the diagram will determine the radius, where 1.0 is the center and 0.50 is the limit of the circumference. The angular location is determined by the number of positive predictions divided by 360 degrees.

**Figure 5:**
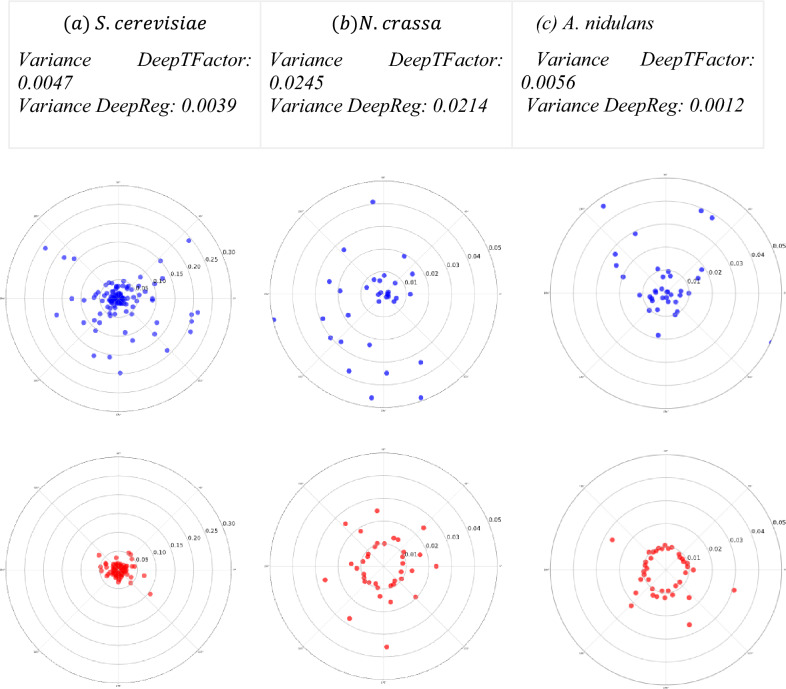
Bull-eye bias-variance tradeoff from true predictions from each model in (**a**) *S. cerevisiae* (**b**) *N. crassa*, and (**c**) *A. nidulans* over experimental values such as ground truth. The red one corresponds to DeepReg, and the blue one is associated with DeepTFactor. Only true positives were considered for this evaluation, where the radius regarding the center is the score obtained and the angle is distributed to fill all the circumference. To show a clear distribution of the points, we cut the diagrams at a certain radius. In supplementary material, Fig. S1, a complete comparison (all predictions per method) is provided.

From this analysis, the DeepReg variance is lower than the DeepTFactor variance, i.e. DeepReg concentrates most of its predictions close to 1, which suggests robustness and quality in our predictions. In addition, it has a lower variance, which suggests that the probability that these predictions to be false positives decreases considerably (Fig. 5). Therefore, the quality and robustness of the predictions could be related to (a) the monitoring of the loss function, avoiding abrupt jumps in it, (b) the hybrid nature of the proposed architecture itself, obtaining the benefits of CNNs and BiLSTM, and (c) the selection of CNN filters useful for finding features.

Therefore, DeepReg excludes possible overfitting by disappearing the effect of the lost function (discontinuous leaps) presented in DeepTFactor Fig. 3. In this regard, DeepReg improves the quality of the predictions with the bias-variance trade off analysis using three reference organisms, where our model presents low variance and low bias Fig. 5. Furthermore, our model improves the performance (F1-Score, precision, and recall), in relation to DeepTFactor Table 1. Finally, DeepReg model identifies almost twice TFs than the DeepTFactor in *S. cerevisiae* Fig. 4.

When we compared our model against TFnet^9^, evident differences emerged. For instance, TFnet uses PSSM matrices as input data to the network. However, the PSSM could include redundant information for proteins from the training set or even, include a bias associated to the domain organization of the TFs, such as the ubiquitous ATP-hydrolysis domain. Therefore, PSSM considers sequence homology, which is precisely what we try to avoid by using DL as a classification tool. Indeed, some sequences could not have a PSSM matrix, because of the lack of similarity with other proteins to construct these matrices. In addition, we consider that metric comparisons, between TFNet and DeepReg, is not reliable, because TFNet metrics on the test set only considers *Homo sapiens* and *E. coli*, whereas our model and DeepTFactor consider proteins from a large diversity of organisms. In addition, the main use of a CNN in TFNet is for conditioning the input to a BiLSTM, whereas the CNN is not used as a feature extractor tool like DeepReg does, since those features are already given by the PSSM.

## Conclusion

In this work, we present a DL model to predict TFs that improves the performance considerably to 0.98 in the F1 score compared to previous models and ML-based models. DeepReg considered two different architectures: CNN and biLSTM, using CNN as a feature extractor and biLSTM as a grammar regulator, and an attention mechanism. Both architectures improved feature extraction by adding specific order filters as well as the selection of the best features by adding an attention mechanism. The best model was selected after performing a series of hundreds of training processes, varying the hyperparameters, and monitoring performance. In general, the DL model identified in average, 71.8% of the experimentally described TFs in three organisms: *S. cerevisiae*, *N. crassa*, and *A. nidulans,* in contrast with DeepTFactor, that identifies in average 64.3% of the experimentally described TFs, in the same models. In addition, the use of the bias-variance tradeoff over the true predictions in DeepReg showed that most of the score predictions were close to 1, i.e. the model exhibits low variance. Finally, the model is robust and reliable in the face of unseen or experimental data, which suggests the elimination of overfitting.

## Supplementary Information

Supplementary Information.

## Data Availability

The datasets analyzed during the current study are available in the Uniprot database, (https://www.uniprot.org/) and in the github https://github.com/LeonardoLed/DeepLearning-_TF section data.
