# Supplementary material for: DeepReg: a deep learning hybrid model for predicting transcription factors in eukaryotic and prokaryotic genomes
Source: Sci Rep. 2024 Apr 21;14:9155. doi: 10.1038/s41598-024-59487-5 (PMC11551149; doi:10.1038/s41598-024-59487-5)
Supplement: Supplementary file 1 — Supplementary Information. [file 41598_2024_59487_MOESM1_ESM.docx]

**Supplementary material**

**DeepReg, a deep learning hybrid model for predicting transcription factors in eukaryotic and prokaryotic genomes**

|  | **TF sequences** | **Non-TF sequences** |
| --- | --- | --- |
| **Amino acid** | **Frequency** | **Frequency** |
| **A** | 14346789 | 543174 |
| **C** | 2238261 | 127245 |
| **D** | 9210935 | 382153 |
| **E** | 11210328 | 533702 |
| **F** | 6615516 | 244172 |
| **G** | 12201403 | 464934 |
| **H** | 3799109 | 205779 |
| **I** | 10191609 | 353133 |
| **K** | 9739927 | 429896 |
| **L** | 16343454 | 666740 |
| **M** | 4156009 | 174059 |
| **N** | 6672399 | 315272 |
| **P** | 7753147 | 421480 |
| **Q** | 6373169 | 359092 |
| **R** | 9337439 | 448417 |
| **S** | 10612839 | 612324 |
| **T** | 8921577 | 386700 |
| **V** | 11802116 | 413335 |
| **W** | 1888028 | 63697 |
| **X** | 7258 | 115 |
| **Y** | 0 | 194741 |

**Table S1. Distribution of amino acids in TF and non-TF sequences**

| **#** | **Definition** | **GO TERMS** | **Functional description** |
| --- | --- | --- | --- |
| **1** | transcription factor | GO:0000976 | transcription regulatory region sequence-specific DNA binding |
| **2** | transcription factor | GO:0000977 | RNA polymerase II transcription regulatory region sequence-specific DNA binding |
| **3** | transcription factor | GO:0000978 | RNA polymerase II cis-regulatory region sequence-specific DNA binding |
| **4** | transcription factor | GO:0000979 | RNA polymerase II core promoter sequence-specific DNA binding |
| **5** | transcription factor | GO:0000981 | DNA-binding transcription factor activity, RNA polymerase II-specific |
| **6** | transcription factor | GO:0000984 | bacterial-type RNA polymerase transcription regulatory region sequence-specific DNA binding |
| **7** | transcription factor | GO:0000985 | bacterial-type RNA polymerase core promoter sequence specific DNA binding |
| **8** | transcription factor | GO:0000986 | bacterial-type cis-regulatory region sequence-specific DNA binding |
| **9** | transcription factor | GO:0000987 | cis-regulatory region sequence-specific DNA binding |
| **10** | transcription factor | GO:0000992 | RNA polymerase III cis-regulatory region sequence specific DNA binding |
| **11** | transcription factor | GO:0000995 | RNA polymerase III general transcription initiation factor activity |
| **12** | transcription factor | GO:0001046 | core promoter sequence-specific DNA binding |
| **13** | transcription factor | GO:0001163 | RNA polymerase I transcription regulatory region sequence-specific DNA binding |
| **14** | transcription factor | GO:0001164 | RNA polymerase I core promoter sequence-specific DNA binding |
| **15** | transcription factor | GO:0001165 | RNA polymerase I cis-regulatory region sequence-specific DNA binding |
| **16** | transcription factor | GO:0001216 | DNA-binding transcription activator activity |
| **17** | transcription factor | GO:0001227 | DNA-binding transcription repressor activity, RNA polymerase II-specific |
| **18** | transcription factor | GO:0003700 | DNA-binding transcription factor activity |
| **19** | transcription factor | GO:0034246 | mitochondrial sequence-specific DNA-binding transcription factor activity |
| **20** | transcription factor | GO:0098531 | ligand-activated transcription factor activity |
| **21** | transcription factor | GO:0106250 | DNA-binding transcription repressor activity, RNA polymerase III-specific |
| **22** | transcription regulation | GO:0001228 | DNA-binding transcription activator activity, RNA polymerase II-specific |
| **23** | transcription regulation | GO:0006351 | transcription, DNA-templated |
| **24** | transcription regulation | GO:0006355 | regulation of transcription, DNA-templated |
| **25** | transcription regulation | GO:0043433 | negative regulation of DNA-binding |
| **26** | transcription factor activity transcription regulation | GO:0045892 | negative regulation of transcription, DNA-templated 19 |
| **27** | transcription regulation | GO:0045893 | positive regulation of transcription, DNA-templated |
| **28** | transcription regulation | GO:0051090 | regulation of DNA-binding transcription factor activity |
| **29** | transcription regulation | GO:0051091 | positive regulation of DNA-binding |
| **30** | transcription factor activity transcription regulation | GO:2000142 | regulation of DNA-templated transcription, initiation |
| **31** | transcription regulation | GO:2000143 | negative regulation of DNA-templated transcription, initiation |
| **32** | transcription regulation | GO:2000144 | positive regulation of DNA-templated transcription, initiation |
| **33** | DNA binding | GO:0003677 | DNA binding |
| **34** | DNA binding | GO:0008301 | DNA binding, bending |
| **35** | DNA binding | GO:0043565 | sequence-specific DNA binding |
| **36** | DNA binding | GO:0050692 | DNA binding domain binding |

**Table S2. GO annotations used to construct the TF database.** The following Gene Ontology annotations were used to retrieve the TFs from SwissProt.

| **Set** | **Accuracy** | **Loss** | **AUC** | **F1-Score** | **MCC** |
| --- | --- | --- | --- | --- | --- |
| Total | 0.9735 | 0.1187 | 0.9696 | 0.9187 | 0.9038 |
| Prokaryote | 0.9803 | 0.0970 | 0.9818 | 0.9485 | 0.9370 |
| Eukaryote | 0.9638 | 0.1547 | 0.9655 | 0.8855 | 0.8661 |

**Table S3. DeepReg Performance on Prokaryotes and Eukaryotes organisms in the validation dataset.**

| **Metrics** | **DeepReg (40000 sequences)** | **Best model in ML (SVM)** |
| --- | --- | --- |
| AUC | 0.9739 | 0.8862 |
| Accuracy | 0.9383 | 0.8920 |
| Precision | 0.9531 | 0.8983 |
| Recall | 0.9263 | 0.8886 |
| Specificity | 0.9511 | 0.8955 |
| F1-Score | 0.9395 | 0.8879 |
| MCC | 0.8769 | 0.7840 |

**Table S4. DeepReg performance with the same data of ML. 40000 sequences were considered to this comparison.**

| $\left( a \right)Saccharomyces cerevisiae$  *Variance DeepTFactor: 0.0232*  *Variance DeepReg: 0.0164* | $(b)Neurospora crassa$  *Variance DeepTFactor: 0.0245*  *Variance DeepReg: 0.0214* | *(c)Aspergillus nidulans*  *Variance DeepTFactor: 0.0234*  *Variance DeepReg: 0.0171* |
| --- | --- | --- |

1.
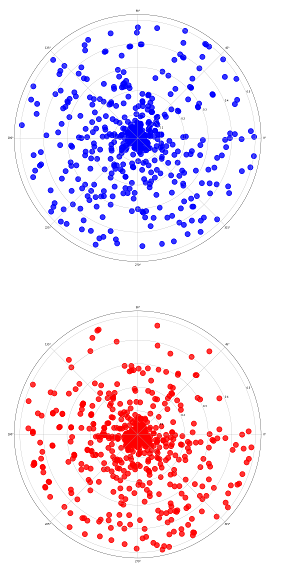

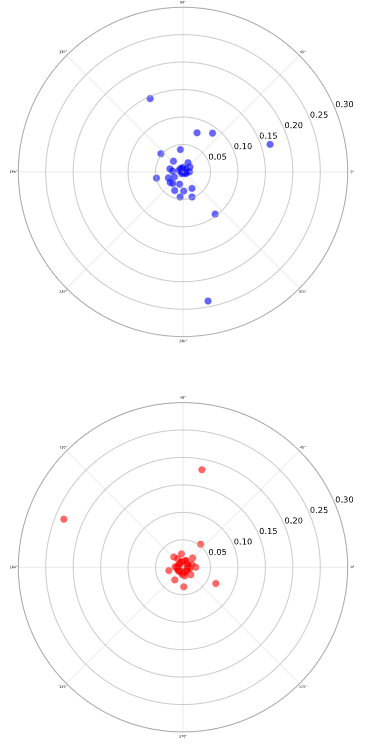

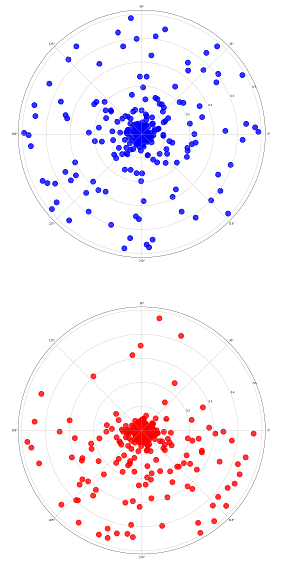
 *(b) (c)*

**Figure S1. Bull-eye bias-variance tradeoff from all true predictions from each model in (a) *Saccharomyces cerevisiae* (b) *Neurospora crassa*, and (c) *Aspergillus nidulans*.** Each point represents a true prediction where the radius with respect to the center is the score obtained and the angle is distributed to fill all the circumference. In blue is indicated DeepTFactor and in red, DeepReg.
